# Perceptions towards online learning among medical students during the COVID-19 pandemic

**DOI:** 10.1016/j.heliyon.2023.e13119

**Published:** 2023-01-21

**Authors:** Qiong Zhang, Qing-zhi Yuan, Peng-qiang Ma, Yue Li, Meng-hui Zhao, Rong-xia Chen, Zhen-gang Tang, Bei Zhang, Bing Liu, Xiang Liu, Fei-feng Li

**Affiliations:** a Hubei Biomedical Detection Sharing Platform in Water Source Area of South to North Water Diversion Project, Hubei University of Medicine, Shiyan, China; b Department of Preventive Medicine, School of Public Health, Hubei University of Medicine, Shiyan, China; c Department of Pharmaceutical Engineering, School of Pharmaceutical Sciences, Hubei University of Medicine, Shiyan, China; d Institute of Neurological Diseases, Shiyan Renmin Hospital, Hubei University of Medicine, Shiyan, China; e Health Management Center, Shiyan Renmin Hospital, Hubei University of Medicine, Shiyan, China

**Keywords:** COVID-19, Online learning, Face-to-face learning, Perceptions, Efficiency of study

## Abstract

Social distancing has been essential during the COVID-19 pandemic to slow the spread of the disease. Online learning ensures students can participate in learning activities while also maintaining a physical distance from other students. Although online learning was used to prevent the spread of COVID-19, the development of online learning has also been promoted. Here, we sought to explore the perceptions and responses of students to online learning during the pandemic using a cross-sectional study. Electronic questionnaire was used for data collection. Statistical analyses were performed for 1614 valid questionnaires and P < 0.05 was considered statistically significant. Overall, COVID-19 had more effect on female students, such as fear of COVID-19 (2.4 times higher than the number of male students) and length of time spent learning (*H* = 42.449, P < 0.05). However, the higher the students’ grades were, the less the impact of COVID-19. For the style of lessons, all students would prefer shorter lessons (P < 0.05). Female and fifth-grade students were more prefer combined online and face-to-face learning, and male and freshmen students were more likely to prefer face-to-face learning after the pandemic. More than 50% of students thought the main advantage of online learning was convenience, with low efficiency being a disadvantage. The main factors negatively influencing online learning were eyestrain, poor network connections, and poor learning environments at home. In conclusion, synchronous online and face-to-face learning may become more common in future curricula, however the efficiency of online learning and the female students more attentions.

## 1 Introduction

COVID-19 is prevalent in more than 200 countries worldwide [1]. To combat the rapidly increasing number of confirmed cases and deaths due to COVID-19, most countries enforced physical distancing measures [2,3]. As a result, education, including higher education, has been greatly affected by COVID-19. In order to continue education, the institutions and students have faced a new challenge of emergency remote learning [4,5]. However, this situation not only creates challenges but also provides the opportunity to promote and develop distance learning [4]. This effect may continue even after the COVID-19 pandemic has passed.

Online learning has been around for a long time [6]. There are numerous types of software and models available for online learning [7]. Some universities around the world had been implementing online learning for a long time prior to COVID-19 [8]. However, online learning has been slow to take off in many countries [8]. The opinions of students are important factors to understand in relation to the benefits and developments of online learning [9]. In this work, we sought to investigate students’ perspectives of online learning during the COVID-19 pandemic.

## 2 Materials and methods

### 2.1 Research objects

We assembled undergraduate students from Hubei University of Medicine, Shiyan, China as a sampling frame for this cross-sectional study. The selection criteria were college students who had go through one semester of online during COVID-19, could engage in barrier-free communication with investigators, and who were willing to cooperate with the project investigators once they understood the reasons for the research. Participants were asked to complete an electronic questionnaire. Ethical approval was granted by the Ethics Committee of Hubei University of Medicine (HUMIRB20210107), and the study met the 1975 Declaration of Helsinki.

Based on the number of indexes, we counted the number of specimens in theory. At last, a total of 1632 questionnaires were retrieved (response rate 32.6%), and 1614 valid questionnaires were used in this work (above the theoretical number). There were 429 males (26.6%) and 1185 females (73.4%). They were from 5 grades (divided according to year) and 16 medical-related majors. The number of freshmen was 49 (3.0%); sophomores 783 (48.4%); juniors 476 (29.5%); seniors 265 (16.4%) and fifth graders 41 (2.5%).

### 2.2 Research tools

To analyze students’ perceptions of the online learning they had already experienced, we collected information from participants in relation to their evaluation and satisfaction of their online learning or lesson. According to methods used in previous studies [10,11], we designed a questionnaire that included questions about basic personal information (gender, grade, major), personal response to the epidemic, basic details of their online learning situation during the COVID-19 pandemic, their opinions about and suggestions for online learning, reasons for choosing traditional face-to-face learning, and potential gains from online learning.

The draft survey instrument was delivered to 6 experts from different specialty (including statistics, preventive medicine and clinical medicine) to assess the validity, reliability and content of the questionnaire. Then a sub-section of the students were selected for a pilot pre-survey prior to the formal survey to test the readability. These students included in piloting the questionnaire were not included in the research and data analysis. Through timely adjustment and modification of the problems identified during the pilot process, the questionnaire and survey methods were further optimized.

### 2.3 Data collection and statistical analysis

To ensure efficiency, an electronic questionnaire was used for this work. All respondents were asked to complete the questionnaire independently by scanning the QR code with mobile phone. To limit any bias in their answers, all participants were informed that the data collection was completely anonymous and that the respondents could not be traced.

A sub-section of the population was selected for a pilot pre-survey prior to the formal survey. Through timely adjustment and modification of the problems identified during the pilot process, the questionnaire and survey methods were further optimized. Statistical analyses were performed using IBM SPSS Statistics software (version 22). The measurement data (continuous variables, also named numerical variables represented by the magnitude of the value) are presented as means±standard deviations and analyzed using an independent-sample *t*-test. Enumeration data (discrete variables, also named categorical variables represented by the form of incompatible categories or attributes) are presented as percentages and analyzed using a chi-square test. P values of less than 0.05 were considered statistically significant.

## 3 Results

### 3.1 Students’ opinions and responses of online learning and COVID-19

We first analyzed the data to look for any differences in the responses of male and female students to COVID-19. A total of 1614 (1185 female, 429 male) students completed the questionnaire, and the female-to-male ratio was 2.8:1. As shown in Table 1, female students were more sensitive to COVID-19; 38.7% of female students expressed a fear of COVID-19, 2.4 times higher than the number of male students (16.1%). Overall, 16.6% of male students expressed no fear of COVID-19, which was 2.7 times higher than the percentage of female students who expressed no fear of COVID-19 (6.1%). This difference was statistically significant (*H* = 100.210, P < 0.05) (Table 1).

**Table 1:** Students’ learning perceptions and states by gender when learning online during the COVID-19 pandemic.

Perception	Degree	Female		Male		Chi-square test results	
		No.	%	No.	%	*H*	P
Fear	0^b^	72	6.1	71	16.6	100.21	8.869 × 10^−21^***^a^
	1	273	23.0	102	23.8		
	2	381	32.2	187	43.6		
	3	429	36.2	65	15.2		
	4	30	2.5	4	0.9		
Learning time	0^c^	64	5.4	58	13.5	42.449	1.346 × 10^−8^***^a^
	1	349	29.5	145	33.8		
	2	226	19.1	63	14.7		
	3	433	36.5	115	26.8		
	4	113	9.5	48	11.2		
Care^d^	0^d^	11	0.9	8	1.9	6.974	0.137
	1	46	3.9	15	3.5		
	2	396	33.4	147	34.3		
	3	588	49.6	192	44.8		
	4	144	12.2	67	15.6		
State^e^	Lowest	142	12.0	46	10.7	0.488	0.783
	Moderate	848	71.6	311	72.5		
	Greatest	195	16.5	72	16.8		

Note: a: *P < 0.05, **P < 0.01, ***P < 0.001; b: 0–4 indicates the degrees of fear of COVID-19, 0 indicates not afraid at all, 4 indicates very scared; c: 0–4 indicates the effects levels of COVID-19 on the length of time students spent studying, 0 indicates no effect at all, 4 indicates very influential; d: “care” means how much did the students attached importance to online learning, 0 indicates no care, 4 indicates very seriously; e: “state” means how did the online learning performance of the students.

We next analyzed the effects of COVID-19 on the length of time students spent studying. COVID-19 had more effect on the length of time female students spent learning, having a major impact on the study time of 65.1% of female students. The figure for male students was 52.7%. This difference was statistically significant (*H* = 42.449, P < 0.05) (Table 1).

More than 90% of the students attached great importance to online learning, and more than 80% of the students had a good online learning status (Table 1). There was no difference between the male and female students (P > 0.05) (Table 1).

### 3.2 The effects of COVID-19 and online learning on students’ academic grades

For students’ academic grades, gender showed a greater influence than the type of learning (online versus face-to-face). As shown in Table 2, for both online and face-to-face learning, female students performed better than male students. In terms of the academic grades of both genders, face-to-face learning results were better than those for online learning (Table 2).

**Table 2:** The effects of COVID-19 and online learning on the academic scores^a^ of male and female students.

Style	Degree	Female		Male		Chi-square test results	
		No.	%	No.	%	*H*	P
Face-to-face learning average score	60–69	132	11.1	78	18.2	18.215	1.858 × 10^−8^***^b^
	70–79	381	32.2	181	42.2		
	80–89	596	50.3	149	34.7		
	More than 90	76	6.4	21	4.9		
Online learning average score	60–69	187	15.8	110	25.6	46.595	4.239 × 10^−10^***^b^
	70–79	346	29.2	157	36.6		
	80–89	572	48.3	129	30.1		
	More than 90	80	6.8	33	7.7		
Chi-square test results	*H*	11.763		11.256		–	
	P	0.008**		0.010*			

Note: a: “academic scores” means the students' academic performance, b: *P < 0.05, **P < 0.01, ***P < 0.001.

### 3.3 Male and female students’ perceptions of online learning

**Fig. 1:**
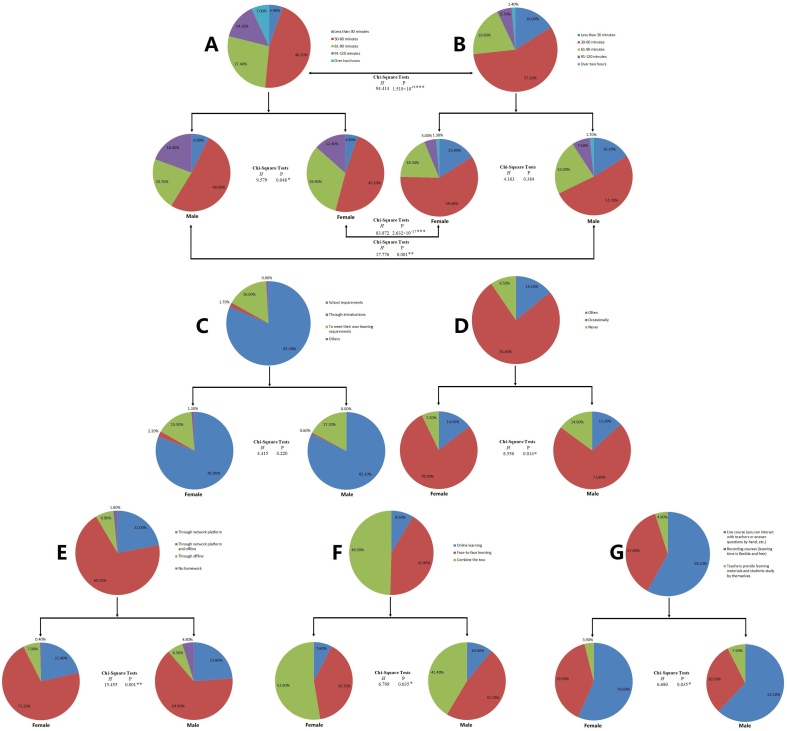
Different perceptions of male and female students in relation to online learning. A: The length of online lessons students have experienced. B: The length of online lessons the students expected. C: The reasons students were exposed to online learning. D: The communication situation among male and female students during the process of online learning. E: Homework completion by male and female students during online learning. F: Course style expected by students in the next semester. G: Style of online learning expected by students in the next semester. *P < 0.05, **P < 0.01, ***P < 0.001.

We analyzed perceptions of the length of lessons of online learning, including the actual and expected lengths of lessons. For the actual length of lessons with online learning, 51.8% of lessons were less than 60 min long (Fig. 1A). However, students would prefer lessons of shorter length (P < 0.05), especially the female students (Fig. 1A and B).

More than 80% of students were exposed to online learning because of their school's requirements, with just 15% of students choosing online learning as it met their own learning requirements (Fig. 1C). During online learning, female students were more willing to communicate with their teachers and classmates (Fig. 1D), and female students were also more likely to complete their homework (Fig. 1E).

In the further study, the female students were more likely to combine online and face-to-face learning following the pandemic (Fig. 1F). However, male students were more likely to prefer face-to-face learning (Fig. 1F). For the style of online learning, most students preferred live courses, so that they could interact with teachers or answer questions by hand (Fig. 1G).

### 3.4 Attitudes of students from different grades towards online learning

For students' responses to COVID-19, in terms of how much they valued online learning and their learning status, there were no differences in students from different grades (Table 3). However, COVID-19 and online learning had a great impact on the study time spent by students of different grades (P < 0.05) (Table 3). In general, the higher the students’ grades were, the less the impact of COVID-19 (Table 3).

**Table 3:** Students’ learning perceptions and states by grade when learning online during the COVID-19 pandemic.

Perception	Degree	Freshmen		Sophomores		Juniors		Seniors		Fifth graders		Chi-square test results	
		No.	%	No.	%	NO.	%	NO.	%	NO.	%	*H*	P
Fear	0^b^	4	8.2	66	8.4	42	8.8	17	6.4	3	7.3	16.992	0.386
	1	11	22.4	187	23.9	109	22.9	68	25.7	10	24.4		
	2	17	34.7	309	39.5	150	31.5	94	35.5	14	34.1		
	3	16	32.7	208	26.6	159	33.4	82	30.9	13	31.7		
	4	1	2.0	13	1.7	16	3.4	4	1.5	1	2.4		
Learning time	0^c^	2	4.1	57	7.3	57	12.0	7	2.6	4	9.8	89.780	2.746 × 10^−12^***^a^
	1	16	32.7	233	29.8	134	28.2	94	35.5	14	34.1		
	2	9	18.4	189	24.1	62	13.0	16	6.0	9	22.0		
	3	20	40.8	237	30.3	168	35.3	112	42.3	8	19.5		
	4	2	4.1	67	8.6	55	11.6	36	13.6	6	14.6		
Care^d^	0^d^	2	4.1	7	0.9	3	0.6	2	0.8	1	2.4	16.572	0.414
	1	2	4.1	31	4.0	21	4.4	6	2.3	2	4.9		
	2	19	38.8	275	35.1	159	33.4	86	32.5	14	34.1		
	3	21	42.9	379	48.4	218	45.8	140	52.8	19	46.3		
	4	5	10.2	91	11.6	75	15.8	31	11.7	5	12.2		
State^e^	Lowest	6	12.2	99	12.6	62	13.0	16	6.0	5	12.2	14.522	0.069
	Moderate	35	71.4	557	71.1	320	67.2	204	77.0	26	63.4		
	Greatest	8	16.3	127	16.2	94	19.7	45	17.0	10	24.4		

Note: a: *P < 0.05, **P < 0.01, ***P < 0.001; b: 0–4 indicates the degrees of fear of COVID-19, 0 indicates not afraid at all, 4 indicates very scared; c: 0–4 indicates the effects levels of COVID-19 on the length of time students spent studying, 0 indicates no effect at all, 4 indicates very influential; d: “care” means how much did the students attached importance to online learning, 0 indicates no care, 4 indicates very seriously; e: “state” means how did the online learning performance of the students.

### 3.5 Different effects of COVID-19 and online learning on the academic scores of students from different grades

As show in Table 4, whether it was via online learning or face-to-face learning, students from different grades also showed different academic performances. On the whole, junior and senior students did best (P < 0.05) (Table 4). Sophomore and junior students achieved higher academic scores through face-to-face learning than online learning (Table 4).

**Table 4:** Different effects of COVID-19 and online learning on the academic scores^a^ of students from different grades.

Style	Degree	Freshmen		Sophomores		Juniors		Seniors		Fifth graders		Chi-square test results	
		NO.	%	NO.	%	NO.	%	NO.	%	NO.	%	*H*	P
Face-to-face learning average score	60–69	4	8.2	143	18.3	47	9.9	10	3.8	9	22.0	91.016	3.138 × 10^−14^***^b^
	70–79	21	42.9	290	37.0	167	35.1	62	23.4	15	36.6		
	80–89	19	38.8	319	40.7	223	46.8	167	63.0	15	36.6		
	Above 90	5	10.2	31	4.0	39	8.2	26	9.8	2	4.9		
Online learning average score	60–69	9	18.4	192	24.5	83	17.4	10	3.8	6	14.6	136.644	2.846 × 10^−23^***^b^
	70–79	19	38.8	272	34.7	148	31.1	44	16.6	14	34.1		
	80–89	16	32.7	272	34.7	201	42.2	188	70.9	19	46.3		
	Above 90	5	10.2	47	6.0	44	9.2	23	8.7	2	4.9		
Chi-square test results	*H*	2.280		14.763		12.558		4.483		1.105		–	
	P	0.516		0.002**		0.006**		0.214		0.776			

Note: a: “academic scores” means the students' academic performance, b: *P < 0.05, **P < 0.01, ***P < 0.001.

### 3.6 Different perceptions of online learning among students from different grades

**Fig. 2:**
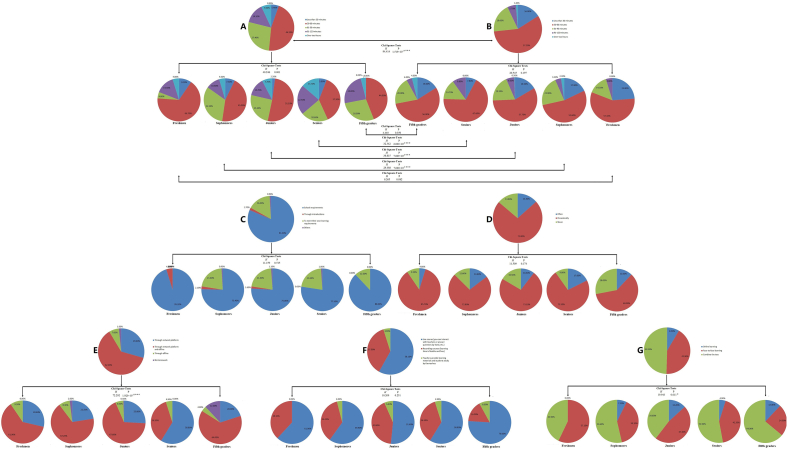
Different perceptions of students from different grades in relation to online learning. A: The length of online lessons students from different grades have experienced. B: The length of online lessons the different grades of students expected. C: The reasons students from different grades were exposed to online learning. D: The communication situation among students of different grades during the process of online learning. E: Homework completion by students from different grades during online learning. F: Course style expected by different grades of students in the next semester. G: Style of online learning expected by different grades of students in the next semester. *P < 0.05, **P < 0.01, ***P < 0.001.

In general, all students wanted class times of shorter length with online learning, regardless of their grade (Fig. 2A and B). The desire for change was higher among sophomore, junior, and senior students (Fig. 2A and B).

There were no differences in the perceptions of students exposed to online learning, their mutual communication during online learning, or their expected style of classes for online learning among students from different grades (Fig. 2C and D and Fig 2F).

In relation to homework during the process of online learning, 62% of students finished their homework either through an online platform or offline (Fig. 2E). In contrast, fifth-grade students had less homework, with 12% of these students stating that they had no homework, which was much higher than the average value (1.6%) (Fig. 2E).

For the arrangement of courses in the next semester, most students (49.5%) stated they would like to combine online learning with face-to-face learning (Fig. 2G). Fifth-grade students in particular preferred a combination of online and face-to-face learning (64.0%) (Fig. 2G). Freshmen tended to prefer face-to-face learning (57.1%), and none of them liked purely online learning (0.0%) (Fig. 2G).

### 3.7 Students’ perceptions of the advantages and disadvantages of online learning

**Fig. 3:**
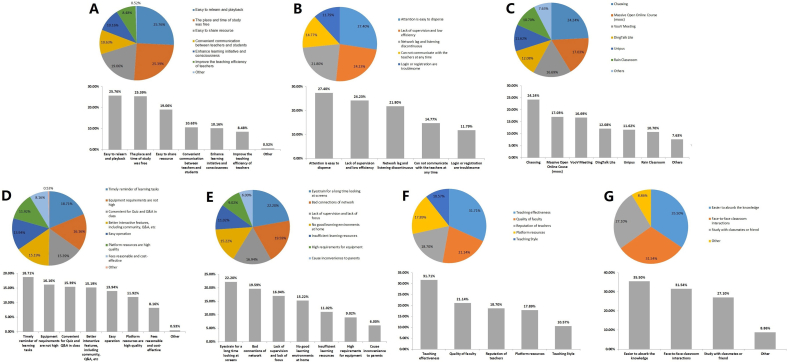
Other perceptions of students about online learning. A: The advantages of online learning recognized by students. B: The disadvantages of online learning recognized by students. C: Software the students have used during their online learning. D: Students' opinions about the ideal online learning platforms. E: Factors affecting online learning efficiency in the students' opinion. F: The aspects of online learning the students were most concerned about. G: Reasons why students want to face to face learning.

We analyzed students’ perceptions of the advantages and disadvantages of online learning. More than 50% of students thought the main advantages of online learning were convenience, such as the convenience of relearning or playing back material (25.76%) and convenience in terms of study time and place (25.39%) (Fig. 3A). For the disadvantages of online learning, most students (51.63%) believed it was the low efficiency, caused by the tendency of their minds to wander (27.40%) and the lack of supervision (24.23%) (Fig. 3B).

### 3.8 Software used during online learning

We found the different types of online learning software were used equally among the students (Fig. 3C). For the characteristic of ideal online learning software/platforms, based on the students’ opinions, they cared more about the convenience of the software/platforms, including timely reminders about learning tasks, convenient operation, and convenient communication (Fig. 3D).

### 3.9 Factors affecting online learning

Finally, we analyzed the factors that affect students' online learning. The main factors influencing online learning were eyestrain, poor network connections, and poor learning environments at home (Fig. 3E). The students’ main concerns about online learning were teaching effectiveness and the quality of the faculty (Fig. 3F). The main reason why they chose to Face to face learning was that it was more convenient to communicate with teachers or classmates and easier to absorb knowledge (Fig. 3G).

## 4 Discussion

Social distancing has been essential during the COVID-19 pandemic to slow the spread of the disease [4,12,13]. Online learning has ensured students could participate in learning activities while also preventing the spread of COVID-19 b y enabling them to maintain physical distancing [14]. The implementation of online learning has effectively helped to prevent the spread of the virus that causes COVID-19 [15]. To some extent, the pandemic has also promoted the development of online learning.

The participants in the teaching process are important factor to achieve the highest educational success, especially with online learning [9,16]. In this work, we chose students who had taken part in online learning and who freely chose to participant in this survey. We analyzed the responses and perceptions of students of different genders and grades in relation to COVID-19 and online learning.

Female students were more sensitive than male students to COVID-19. The pandemic also had a greater impact on the learning time invested by female students in online learning. For female students, their online learning performance was not as good as face-to-face learning. However, there were no differences in how much they cared or the learning state between female and male students in the process of online learning. It has been found that the knowledge, attitudes, and practices (KAP) scores showed no significant difference among female and male students [17]. Counterparts also revealed that half of female students have higher anxiety states and lower sleep quality, and like people with COVID-19 infection, females had higher rates of anxiety, depression, sleep problems during the pandemic [18]. We should consider providing more psychological support and care for female students when they are learning online during a pandemic period.

There were no differences in the response to COVID-19 between students from different grades. However, the pandemic had a greater impact on the learning time invested by students from lower grades. It may be that seniors are more adaptable and less affected by COVID-19 and online learning.

Student attitudes and ease of access to online learning are positive factors for its promotion [19,20]. Compared with face-to-face study, some students also find it easier to participate in online learning [21]. According to our results, the lesson length and duration of online learning should be moderate (such as less than 60 min or less). More than 20% of students expressed that looking at a screen for prolonged periods caused eyestrain that hindered their studies. Although the length of lessons the students took varied from grade to grade, the lesson length they preferred was remarkably similar. Most students preferred lessons that lasted between 30 and 60 min, and more than 90% of the students preferred lessons of less than 90 min.

Many students believed that online learning was useful during the COVID-19 pandemic [22]; however, they prefer normal semester learning [10,23]. Here, we found that students would prefer a combination of online and face-to-face learning in their future studies, especially female and higher grade students. For the form of online learning, most students preferred “live courses”, in which they can interact with others. Other researchers also found that synchronous online and face-to-face learning are used most during normal semester learning [23,24]. So, this way of learning may become an increasing trend in future curricula, even beyond the COVID-19 pandemic.

Online learning has attracted global attention [25,26], and there are many types of software and modes for online learning [27]. Recorded broadcast courses and massive open online courses (MOOCs) are the online learning software types or modes that students are most familiar with [28]. At the same time, students are increasingly using asynchronous online courses, because they can play back and watch the teaching videos at any time [29]. In this study, we also found that in the opinions of students the advantages of online learning were the ease of play back and relearning (25.76%) and freedom to choose the time and place of learning (25.39%).

However, the main disadvantage of online learning was low efficiency because it was easy to become distracted (27.40%) and because of the lack of supervision (24.23%). The main task of instructional designers is to adopt approaches in which teaching efficiency can be controlled [28]. For the students, the aspects of online learning they were most concerned about were teaching efficiency (31.71%) and the quality of the faculty (21.14%). Most students chose face-to-face learning because it was more conducive to knowledge absorption. Others researchers have also found that knowledge absorption and the effectiveness of students when learning online should be considered at all stages of online learning [30,31]. So, during the process of online teaching, educators should pay more attention to the efficiency of students’ learning.

In this work we studied the perceptions and responses of students to online learning and COVID-19. Our results implied that synchronous online learning and face-to-face learning may become an increasing trend in future curricula even beyond the COVID-19 pandemic. In order to improve the efficiency of online learning, we should take measures including limiting the length or duration of online learning lessons, improving the convenience of learning, and enhancing communication during online learning. Especially, the female students required more psychological intervention and care when engaged in online learning during the epidemic period. All of those were important for the benefits and developments of online learning.

## Author contributions

Conceived and designed the experiments: FF L, Q Z, X L; Performed the experiments: QZ Y, PQ M, Y L, MH Z, RX C, FF L; Analyzed and interpreted the data: FF L, PQ M, B L; Contributed reagents, materials, analysis tools or data: FF L, Q Z, X L, ZG T, B Z, QZ Y, B L; Wrote the paper: all authors.

## Declaration of competing interest

All authors declare that no competing interests exist.

## Financial disclosures

There are no financial disclosures from any authors.

## Funding statement

This work was supported by grants from the Cultivating Project for Young Scholars at 10.13039/501100014361Hubei University of Medicine (2020QDJZR025); College Students Innovation and Entrepreneurship Training Program at 10.13039/501100014361Hubei University of Medicine (*X*202110929005, *X*202110929007); Medical Education Branch of the Chinese Medical Association, 2020 Medical Education Research Project of Medical Education Professional Committee of the Chinese Higher Education Association (2020B-N12302); Hubei Education Science Planning Project (2021ZA06); and Teaching Research Project of Hubei University of Medicine (2021015, 2021023). The funders played no role in the study design, data collection and analysis, decision to publish, or preparation of the manuscript.
